# In Vitro Models of the Blood–Cerebrospinal Fluid Barrier and Their Applications in the Development and Research of (Neuro)Pharmaceuticals

**DOI:** 10.3390/pharmaceutics14081729

**Published:** 2022-08-18

**Authors:** Fatemeh Dabbagh, Horst Schroten, Christian Schwerk

**Affiliations:** Department of Pediatrics, Pediatric Infectious Diseases, Medical Faculty Mannheim, Heidelberg University, 68167 Mannheim, Germany

**Keywords:** BCSFB, blood–cerebrospinal fluid barrier, choroid plexus, drug permeability, drugs, in vitro model, therapeutics

## Abstract

The pharmaceutical research sector has been facing the challenge of neurotherapeutics development and its inherited high-risk and high-failure-rate nature for decades. This hurdle is partly attributable to the presence of brain barriers, considered both as obstacles and opportunities for the entry of drug substances. The blood–cerebrospinal fluid (CSF) barrier (BCSFB), an under-studied brain barrier site compared to the blood–brain barrier (BBB), can be considered a potential therapeutic target to improve the delivery of CNS therapeutics and provide brain protection measures. Therefore, leveraging robust and authentic in vitro models of the BCSFB can diminish the time and effort spent on unproductive or redundant development activities by a preliminary assessment of the desired physiochemical behavior of an agent toward this barrier. To this end, the current review summarizes the efforts and progresses made to this research area with a notable focus on the attribution of these models and applied techniques to the pharmaceutical sector and the development of neuropharmacological therapeutics and diagnostics. A survey of available in vitro models, with their advantages and limitations and cell lines in hand will be provided, followed by highlighting the potential applications of such models in the (neuro)therapeutics discovery and development pipelines.

## 1. Background

The research and development platforms of therapeutics targeted to the brain or aimed to be excluded from the central nervous system (CNS) depends mainly on their accomplishment in assessing the reciprocal behavior of brain barriers and the pharmacologically active agent. This effort is made to conquer the economic and technical burden on the pharmaceutical industry attributable to high risk and poor approval rates of neurotherapeutics. During the long journey of drug development, based on the nature of the product under study and in the cases of pregnant women, neonates, and any conditions with the possibility that drugs might have short- or long-term damaging effects, substantial data are required to prove that a substance is excluded effectively from the brain. On the other hand, when the aim is efficient drug delivery to the CNS, the development of neurotherapeutics faces a number of major challenges, with difficulty in delivering them to their desired site of action in the brain being on top. The presence of highly selective barriers (the blood–brain barrier (BBB) and the blood–cerebrospinal fluid (CSF) barrier (BCSFB)) and the complexity of the highly regulated brain environment are among the factors responsible for the elevated attrition percentage during the development of these CNS-targeting medicines.

The BBB and BCSFB are of utmost importance to pharmaceutical drug discovery, as both barriers provide obstacles to the penetration and delivery of therapeutics and diagnostics designed for the management of CNS complications and disorders. Presently, leading research has been concentrated on the brain vascular endothelium as a medicinal target to improve the delivery of brain therapeutics. However, the more poorly noticed BCSFB is also an essential gateway to the CNS, since it is broadly accepted that most substances, even macromolecules, that successfully reach the CSF, can find their way further to the brain parenchyma. Taken together with its considerable surface area, which is estimated to be ~25 to 50% the size of the inner capillary surface area of the brain [1], the BCSFB can be regarded as a target for the CNS delivery of (neuro)therapeutics [2,3]. Notably, the unique role of this barrier to regulate the composition of the CSF offers the BCSFB also the privilege of augmenting the levels of certain compounds and drug candidates in the CSF to accomplish therapeutic benefits and availability to the brain parenchyma.

Species relevant in vitro model systems of the BCSFB play an essential role in enabling the understanding of this barrier’s properties and developing potential interventional techniques and therapeutics. In the pharmaceutical, pharmacological, and toxicological fields, these models harbor the potential to be applied for investigational drugs/compounds screenings, permeability and transport assays, (neuro)toxicological evaluations, and mechanistic molecular pharmacological studies and related assays at the pre-clinical level. Due to many economic concerns, ethical issues, and the necessity to comply with the three R rules (the replacement, reduction, and refinement of animal subject experiments) and bypassing animal testing as far as possible, there has been a great effort in past decades toward establishing robust and convenient models to study basic characteristics of the barriers and also to facilitate the research and pre-clinical investigations of neurotherapeutics in the laboratory under controlled conditions. Such models should authentically mimic the in vivo microenvironment phenotype of the BCSFB and demonstrate representative properties of functional elements of tight junctions (TJs) leading to high transepithelial electrical resistance (TEER), restricted paracellular permeability, low non-specific pinocytic activity, and the expression of receptors and transporters.

With regard to the brief introduction, and despite the challenges and drawbacks inherited by these in vitro model systems, it is noticeable that the area has gained significant attention over the previous decades. These in vitro platforms offer a less resource-intensive alternative approach compared to animal models, in addition to an ease of manipulation, which gives rise to their exploitation during high-throughput end-point drug screenings for the development of drug candidates, and also for providing insights into the mechanistic principles governing CNS drug delivery. Furthermore, such models can be helpful to enlighten the principal translocation routes of small-molecule drugs and constantly increasing number of biopharmaceuticals. To this end, the present review sets out to summarize the efforts and progresses made to this research area with a notable focus on the attribution of these models and techniques to the pharmaceutical sector and the development of neuropharmacological therapeutic and diagnostic agents. It is hoped that researchers new to the field find this paper to be a straightforward guide to figure out the suitable in vitro models and optimal techniques for their particular interest.

## 2. Structure and Physio-Anatomical Features of the BCSFB

A more precise understanding of the BCSFBs physiological and anatomical attributes will pave the way toward the rational development of CNS-targeting small and large molecule therapies, aiming to treat a wide range of neurological conditions. The structure and physio-anatomical features of the BCSFB have been the subject of numerous excellent and comprehensive reviews [1,4,5,6,7,8], so only those features relevant to pharmaceutical/pharmacological research and neurotherapeutics development will be briefly discussed here.

**Figure 1:**
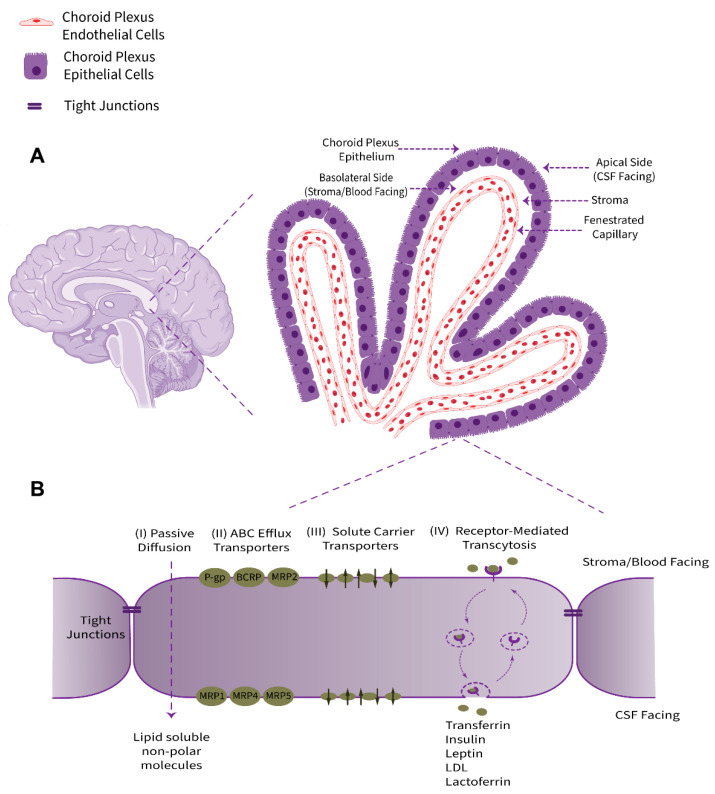
Schematic representation of anatomical location, physiological properties, and pharmacologically related transport systems of the BCSFB. (**A**) The BCSFB structure is comprised of the choroid plexus polarized cuboidal epithelial cells surrounding the highly permeable fenestrated capillaries of stromal core and tight junctional strands uniting adjacent epithelial cells. The innermost capillary (containing red blood cells), with leaky inter-endothelial gap junctions, is alongside the underlying stroma/basement membrane extracellular matrix. The epithelial basolateral surface faces stroma/blood and is in contact with interstitial fluid (ISF). The brush border apical membrane containing microvilli faces the adjacent CSF. (**B**) The main transport-relevant features of the BCSFB in terms of influx and efflux transport systems responsible for supplying nutrients, hormones, and therapeutics to the brain/CSF or acting to eliminate metabolites, xenobiotics, and neurotoxic compounds, respectively, are depicted.

The choroid plexus, as a villous connective and highly vascularized structure, is the major component of the BCSFB system that connects the two principal physiological circulatory systems, namely the peripheral blood circulation and the CSF bulk flow. The choroid plexus convolute consists of two tissue layers, an outer secretory roughly cuboidal epithelium and an inner underlying stromal core comprising an immense network of fenestrated leaky blood vessels with a rich extracellular matrix. Cellular and sub-cellular foundations of choroid plexus barrier properties are non-neuronal epithelial cells (defined as a subtype of macroglia, derived from neuroectoderm) and their adjacent TJs (Figure 1). From an anatomical point of view, this ventricular structure is protruded in the brain lateral, third, and fourth ventricles. The choroid plexus epithelium is in line with the ependyma, a cuboidal epithelium interconnected by gap junctions covering the lumen of the cerebral ventricles; however, despite sharing a common embryological origin, these two cell types are quite distinct [9].

The choroid plexus contributes to the production and secretion of CSF (approximately 500 mL per day in humans, or more precisely ~0.4 mL/min/g choroid plexus in adult mammals) and cerebral homeostasis by regulation of the blood–CSF exchange taking advantage of numerous transport systems allocated in a polarized configuration among the apical or brush border (luminal, CSF-facing) and the basolateral (abluminal, stroma-, or blood-facing) membranes of the choroidal epithelial cells [10]. The choroid plexus epithelium supplies micronutrients, hormones, growth factors, neurotrophins, and neuroprotective proteins to the CSF–brain nexus and uniquely provides micronutrients, such as ascorbic acid (vitamin C) and folate, for neuronal networks and glia [11,12,13,14]. Taken together, the choroid plexus acts in a complementary collaboration with brain microvessels to furnish regulatory factors and essential substances to meet the cerebral metabolism requirements.

### 2.1. Tight Junctions

As a formidable structural and metabolic barrier, the BCSFB, similar to the BBB, prevents toxic substances in the circulation from reaching the brain. The structural elements of this barrier are dense bands of impermeable TJs near the apical (CSF-facing) surface of adjacent cells of the choroid plexus epithelium. TJs are elaborate networks of specialized membrane microdomains, which tighten adjacent epithelial cells together and consequently limit the free diffusion of hydrophilic and polar molecules. Morphologically and structurally, TJ complexes comprise transmembrane (integral) proteins as occludin, the claudins, or junctional adhesion molecules (JAMs), and the associated cytoplasmic proteins of the membrane-associated guanylate kinase-like homologues (MAGUKs) family, termed zonula occludens proteins ZO-1, ZO-2, and ZO-3. Non-MAGUK peripheral junction proteins, such as cingulin, AF-6, symplekin, 7H6, and 4.1R have also been shown to be associated with TJs [15]. Claudins 1, 2, 3, and 11 have been described as the dominant members of this family present in choroid plexus epithelial cells [3].

### 2.2. Specific Markers and Receptors

A large number of transport proteins and receptors are expressed in choroid plexus epithelial cells for mediating and controlling multiple functions of this tissue and acting to transduce key humoral signals between the blood and the CNS. For instance, the choroid plexus serves to transport an assortment of amino acids bidirectionally (e.g., glycine, alanine), hormones (growth hormone, thyroid hormones, insulin, and melatonin), proteins, and growth factors (transthyretin or TTR, transferrin, prolactin, vasopressin, leptin, insulin-like growth factor 1 and 2, or IGF-1 and IGF-2, respectively) and pharmacologically active agents (e.g., β-lactam antibiotics, cimetidine, and benzylpenicillin) [16]. The choroid plexus also expresses high levels of receptors for low-density lipoprotein (LDLR), LDLR-related protein 1 and 2 (LRP1 and LRP2), endothelin, serotonin (5-HT), formylpeptide receptor-like 1 (FPRL1), arginine vasopressin (AVP), and atrial natriuretic peptide (ANP) [17,18,19]. Among the markers mentioned above, transthyretin is considered a specialized feature of choroid plexus epithelial cells.

### 2.3. Transporters and Ion Channels

Choroid plexus epithelial cells possess multiple structural and functional attributes of a transporting epithelium and express a broad spectrum of uni- and bi-directional transporters and ion channels, distributed in a polarized fashion at the apical and basolateral surfaces. This polarized distribution between luminal/apical and abluminal/basolateral membranes enables a heavy yet regulated and directional movement of molecules/compounds across the BCSFB. Multiple transporter proteins, classified as solute carrier (SLC) transporters, are present and functional at the epithelial cells of the BCSFB for glucose, amino acids (acidic, basic, and neutral), peptides, organic anions, monocarboxylic acids, and a myriad of solutes and ions [20,21,22,23,24,25,26,27,28,29,30,31,32]. A summarized list of SLC transporters at the BCSFB is provided in Table 1.

### 2.4. Xeno- and Endobiotic Efflux Systems

One vital physiological commitment of the choroid plexus is to furnish effectual clearance of xenobiotics and potentially deleterious metabolic end products out of the CSF back to the circulatory system, and the choroid plexus is consequently nominated as kidney of the brain [33]. This aim is achieved through the high capacity of the choroid plexus for drug metabolism using metabolizing (reducing, hydrolyzing, or conjugating) enzymes and the existence of specialized transporters.

The principal choroidal drug transporters accepting a broad range of substrates belong to two superfamilies of transporters, namely ATP-binding cassette (ABC) carrier transporters and the SLC family [34]. The superfamily of ABC transporters functions as ATP-driven efflux pumps, playing an essential role in the barrier functionality of the BCSFB. The human genome embraces 49 genes encoding ABC transporters, arranged in distinct subfamilies of A to G. The three subfamilies B, C, and G contain transporters that essentially handle xenobiotics and are expressed to a greater extent in the barrier and excretory tissues compared to all other cells [35]. From a pharmacological point of view, these transporters can be regarded as a double sword, being neuro- and xeno-protective on one hand, when they limit neurotoxicants entry into the brain, whereas they might hinder effective drug delivery for CNS pharmacotherapy on the other hand. Multiple ABC transporters are expressed in the choroid plexus epithelium, playing a pivotal role in CNS therapeutics delivery, and absorption, distribution, metabolism, and excretion (ADME) of drugs. ABCC1/MRP1, ABCC4/MRP4, and ABCC5/MRP5 are located in the basolateral membranes, enabling active transport from the cytosol toward underlying fenestrated capillaries, while the efflux pump P-glycoprotein (P-gp/ABCB1/MDR1), ABCC2/MRP2, and ABCG2/BCRP are positioned in the apical membrane, allowing transport from the cytosol toward the CSF [28,36,37,38,39,40,41,42,43].

In addition to the aforementioned efflux pumps expressed at luminal and/or basolateral membranes, diverse drug-metabolizing enzymes, responsible for inactivating endogenous and exogenous molecules, are expressed in the choroid plexus, including glutathione S-transferases (GSTs), UDP-glucuronosyltransferases (UGTs), flavin-containing monooxygenases (FMOs), epoxide hydrolases (EHs), and phase I metabolizing enzymes, namely multiple isoforms of the cytochrome P450 family [4,41,44,45,46,47,48,49,50,51].

## 3. Survey of Available Platforms

**Figure 2:**
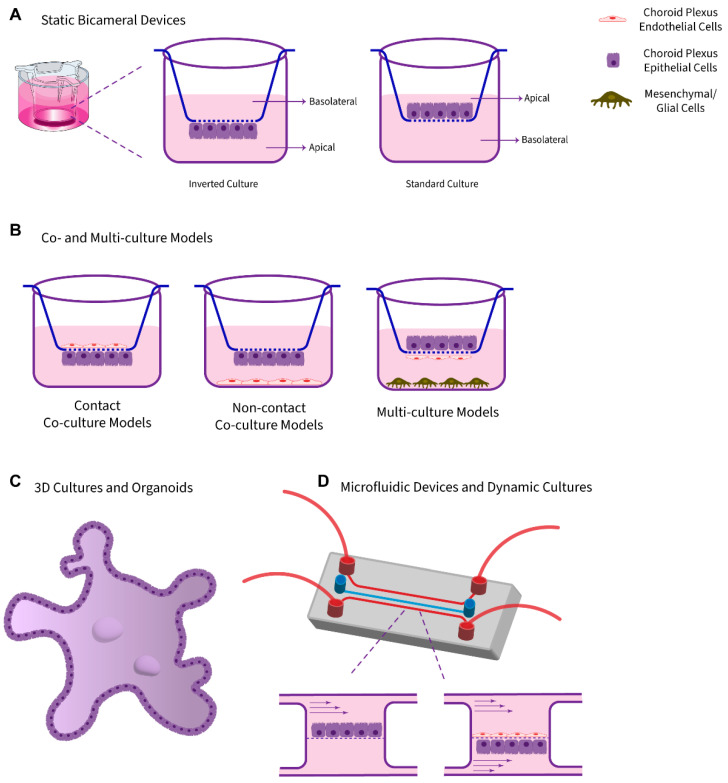
Various possible in vitro BCSFB model platforms are schematically depicted here. (**A**) Static bicameral devices or alternatively known as cell culture filter inserts monolayers. This compartmentalized model, as the mostly utilized configuration by a preponderance of studies, represents culture of choroid plexus epithelial cells either in standard or inverted format on a suitable permeable filter insert; (**B**) Co-culture and multi-culture filter inserts. Here, choroid plexus epithelial cells are grown on porous cell culture inserts alongside endothelial, mesenchymal (e.g., pericytes), and/or glial cells either cultivated into the bottom of a multi-well plate in which the insert is located (non-contact) or seeded on the opposite side of the inserts containing epithelial cells (leading to a so-called back-to-back contact co-culture); (**C**) Three-dimensional cultures and organoids; (**D**) Dynamic cultures or microfluidic devices. The upper and lower channels of the devices are separated to model the luminal and abluminal membrane interfaces based on cell culture direction and a tunable shear stress is induced by a continuous flow of culture medium.

Given the principal role of the BCSFB and restrictions of animal and human investigations, there is a continually increasing demand for new and advanced model systems and in vitro techniques. The following sections describe and summarize the current knowledge related to in vitro models of the BCSFB and focus on their relevance to (neuro)therapeutics research and development. These in vitro model systems are arranged in a diverse range of technical, biological, and physiological complexities (Figure 2). It should be commented that no single model is a perfect representation of the in vivo complexity, and each model inherits pros and cons, which should be considered upon investigations (Table 2).

### 3.1. Static Monolayer Cultures Using Bicameral Systems

Basic 2D models are static systems of choroid plexus epithelial cell monocultures, predominantly based on semipermeable microporous membranes. The static culture systems are defined as those in which cultured cells are kept in the absence of physiological fluid dynamics. In this context of negligible fluid shear stress, the exchange of nutrients and waste takes place using diffusion [52]. These in vitro BCSFB epithelial barrier models have conventionally been founded on bicameral systems (alternatively called cell culture inserts), which possess a cell culture support fabricated from microporous permeable polymer membrane, generating distinct environments on opposing surfaces of a cell monolayer [53]. Upon cultivation, choroidal epithelial cells usually form a tight and polarized confluent monolayer, which can be regarded as a physiologically active cell culture model of the blood–CSF barrier. If cells are grown on top of the semipermeable membranes, this system’s upper or apical compartment simulates the ventricular space, and the basolateral/lower reservoir mimics the blood/stroma side. In the case of the inverted culture of epithelial cells on the lower face of inserts, such as in the method described by Dinner et al. [54], the apical and basolateral directions would be switched. In this manner, either transfer directions (blood-to-CSF and CSF-to-blood) are easily and independently accessible for further analyses.

The facile processing of filters makes this system an appropriate candidate for high-throughput screening (HTS) studies of drug permeability, binding affinity measurements, and transport kinetics. In general, this model type is well suited to explore in detail transport mechanisms (ranging from passive diffusion, facilitated transport, receptor-mediated transport) and cellular transmigration processes, and to investigate the permeability of potential drug candidates as well as the transepithelial resistance. Other applications include the analysis of the metabolism of molecules en passage and the determination of the ensuing metabolites [10]. Based on an experimental design proposed by Strazielle and Preston [10], these model systems can be used to confirm the active transport of an investigational compound, by observing an imbalance across both compartments containing an initial equimolar concentration of the compound and its accumulation against a concentration gradient.

In spite of the advantages pertained to the ease of manipulations of static bicameral model systems, there are disadvantages mainly due to the two-dimensional nature, the absence of fluid-induced shear stress, and the lack of fundamental features, as 3D structure, cell–cell or cell–matrix interactions, comparable TEER, fluid-induced shear stress, vasculature, and the presence of other accompanying cells, necessary to establish a flawless and authentic in vitro model [55]. The “edge effect” arising from the higher permeability of inserts at the outer rim of the membrane is another drawback that should be considered [56]. In addition, cell culture insert-based model designs are not specialized for microscopy and, therefore, do not provide an ideal platform for direct visual inspection of pharmaceuticals’ transport behavior and mechanisms. According to Larsen et al. [53], the main technical hindrance lies in the distance separating the cell monolayer and the below chamber that is not in compliance with the working distance of high magnification, high numerical aperture objectives. As an alternative solution, excised fixed semi-permeable membranes can be mounted on coverslips for imaging; still, this approach provides static snapshots of the transport status and is incapable of capturing the dynamics of (drug)substances’ cellular trafficking. However, commercially available image-compatible platforms can circumvent this obstacle and facilitate high-resolution (live)-cell imaging.

Special attention should be taken for the proper selection of the type of culture inserts for the experiments and the coating of filters prior to cells seeding.

Selection of the culture insert: the choice of the cell culture insert depends on the application and desired outcomes of the study. A broad range of inserts with varying permeable membrane materials (polycarbonate (PC), polyester polyethylene terephthalate (PET), and collagen-coated polytetrafluoroethylene (PTFE)) and different porosities (pore size and pore density) are available commercially from several manufacturers. The selection of the cell culture insert is a crucial step in the design of preliminary experiments. It is recommended that the adsorption and retainment profile of investigational compounds to different types of filter materials are clarified in each experimental setting. The optical properties of filters should also be considered. Transparent membranes offer the advantage of assessing cell culture monolayers using phase-contrast microscopy. A 0.4 µm pore size is well suited to examine the transfer of solutes and a large number of proteins. Theoretically, free diffusion is not restricted in the case that the pore diameter is wider than 20-fold the effective diameter of the compound [10].

Coating material: precoating the semi-permeable membrane support using different basal lamina components and comparable compounds (laminin, collagen, fibronectin, Matrigel^™^, and poly-D-lysine) can enhance cell attachment, growth, and spreading to form a confluent monolayer. As the most appropriate matrix component for culturing choroid plexus epithelial cells, laminin is considered the principal glycoprotein component of the basement membrane serving to expedite cell attachment, spreading, and growth [57]. It was highlighted that a laminin coating ensured the formation of confluent monolayers of porcine choroid plexus epithelial cells, owing to improved adherence and proliferation [58]. Alternatively, collagen is more cost-effective yet still equally efficient. Nevertheless, collagen might contribute to enhancing the adhesion and growth of fibroblasts available in the cell suspension, consequently disturbing the purity of the cultures [59].

### 3.2. Co-Culture Models

To generate a model resembling more closely the in vivo scenario, possessing functional TJs, high TEER, and expression of specific transporters and enzymes, co-culture systems can be developed [60,61,62,63,64]. To enhance the quality of BCSFB in vitro models, cell culture barrier models can be configured in complex co-culture systems, in which choroid plexus epithelial cells are grown on porous cell culture inserts alongside endothelial cells, mesenchymal (e.g., pericytes), and/or glial cells either cultivated on the bottom of a multi-well plate into which the insert is located (non-contact) or seeded on the opposite side of the inserts containing epithelial cells (leading to a so-called back-to-back contact co-culture).

A co-culture model of choroid plexus epithelial and endothelial cells cultivated on opposing surfaces of cell culture filter inserts has recently been described [65]. To our knowledge, it is the first report of establishing a co-culture in vitro model of the BCSFB. Introducing the vascular component to the model systems, not only results in an upgrade of the model to a more physiologically related one, but also can provide a platform to study the emerging roles of the choroid plexus vasculature in organ function. This can further pave the way for studying dysregulated interactions, such as the gut–brain axis and even clarifying therapeutic targets in various disorders [66]. Despite exhibiting often excellent barrier properties and a more resemblance to an in vivo phenotype, co-culture models are in general labor- and capital-intensive.

### 3.3. 3D Cultures and Organoids

State-of-the-art three-dimensional (3D) culture systems, including matrix-based and matrix-free models, such as organoids and spheroids, have been recognized as the next advancement from static mono- and co-cultures. These 3D platforms with diverse features constitute promising alternatives to animal models and 2D cell culture systems in an in vitro tool to recapitulate the complex features of cerebral barriers [67,68]. In order to recapitulate the choroid plexus cytoarchitectural arrangement and a model resembling physiological conditions more closely, 3D cultures and organoids can be approached. Compared to other, less sophisticated in vitro counterparts, these models most accurately reflect the BCSFB properties and are valuable alternative tools to the use of animal subjects in CNS-oriented drug discovery programs.

#### 3.3.1. 3D Explants and Cultured Cells in a Scaffold System

The simple design of appropriate cells cultured in a suitable scaffold or gel system, can be instrumental in the generation of high-throughput in vitro BCSFB models. In spite of few experimental experiences with the BCSFB, the currently available knowledge of BBB cells cultured in 3D platforms can be extended to the BCSFB field [69,70,71]. Three-dimensional explants of the choroid plexus can be generated from fragments of choroid plexuses, dissected from animals, human postmortem tissue, or human surgical samples cultured in suitable matrices, such as Matrigel^™^ and other commercially available hydrogels [72,73]. Three-dimensional explant platforms of mouse, rat, and human choroid plexus have been well prepared and maintained in the culture [72,74,75,76].

#### 3.3.2. Organoids and Self-Organized 3D Models

Organoids, as defined by Huch et al., are 3D structures derived from either pluripotent stem cells or neonatal or adult stem/progenitor cells, in which cells spontaneously self-organize into properly differentiated functional cell types, and which recapitulates at least some function of the organ [77]. In the case of brain organoids, these accurate and versatile in vitro models reproduce several attributes of the brain barriers, including the expression of tight junctions, molecular transporters, and drug efflux pumps, and are critical tools for the study of brain barriers transport and the development of theranostics that can reach the CNS [78,79]. The potential to be scaled up to a high-throughput format, the ease of culture, and the miniature size nominate these multicellular organoids as robust, reliable, and predictive platforms to analyze and screen brain-penetrating compounds for the discovery of new and optimized treatment approaches for various neuropathologies.

Organoids of the choroid plexus as in vitro research platforms, derived from human pluripotent stem cells (human embryonic stem cells (ESCs) and induced pluripotent stem cells (iPSCs)) recapitulate fundamental morphological and functional attributes of this organ [80]. These sophisticated models overcome the disadvantage of species-to-species differences and can improve our understanding of the development/function of the human choroid plexus, which due to the lack of experimental access to this vital brain tissue, is still elusive. A pluripotent stem-cell-derived organoid model of the choroid plexus has been developed by Pellegrini et al. [81,82], which brings about a tight barrier and reliably exhibits features of CSF production/secretion and the selective transport of molecules. According to the authors, this model could predict the permeability of pharmacologically relevant compounds qualitatively and quantitatively.

The aforementioned organoid models are devoid of vasculature, which can be regarded as both an advantage (in case of isolated investigations in a context free of interfering secondary complications) or a disadvantage (resulting in limited oxygen and nutrient delivery to the inner-most regions) depending on the study purpose. Despite the apparent drawbacks of lacking fluid flow-induced shear stress and burdensome establishment, this model platform is cost-effective, reproducible, and can serve as a pre-clinical array to narrow the translational gap between animals (mainly rodents) and human clinical trials. As a pre-clinical model scheme, patient-derived organoids may also pave the way toward the individualized translation of therapeutics [83]. Hypothetically, these 3D in vitro models offer an acceptable homogeneity and complexity level, enabling low- to a medium-throughput screening of a substantial number of investigational compounds to be evaluated in the process of novel therapeutics development. Other potential applications embrace evaluation of the (neuro)toxicological profiles of investigational compounds, lead compound identification/optimization phase studies, and structure–activity relationships (SAR) analyses. Taking advantage of automated microscopy and robotic-assisted technologies, the throughput of this model platform can be further enhanced [56].

#### 3.3.3. Three-Dimensional Bioprinting Strategies

Three-dimensional bioprinting, as an additive manufacturing technology for modeling of user-defined biological samples, has emerged as a promising tool for the expansion of the BBB models. There is no known report of the in vitro 3D-bioprinted models of the BCSFB until now, but there are studied cases of the BBB [84,85,86,87,88], which have the potential to be adapted by the researchers in the field of the BCSFB. In principle, major printing modalities of inkjet-based, extrusion-based, and light-assisted bioprinting can be exploited to establish models with a high level of heterogeneity and biomimicry, which possess great potential as drug screening platforms [84]. Indeed, 3D printed models provide advantages of uniform and reproducible manufacture, minimal operating time, diminished user error, precisely controlled size, flexibility, and a high throughput compared to traditional techniques [89].

### 3.4. Dynamic Models and Microfluidic Platforms

To generate experimental conditions largely comparable to the in vivo environment, the impact of hydrostatic pressure and fluid-induced shear stress can be incorporated into in vitro model platforms. In the light of this phenomenon and with further technological advances, in addition to static models, dynamic systems and culture in microfluidic chamber devices have been evolved, in which a tunable shear stress is induced by a continuous flow of culture medium, in either pump-based or pumpless dynamic configurations [52].

Microfluidic device-based models, or so-called organ-on-a-chip systems, are dynamic models with a precisely controlled periodic physiological fluid flow that tend to augment the survival of three-dimensional cultures and organoid models, with improved nutrients/wastes and oxygen exchange, and more realistic dimensions and geometries. Microfluidics-based approaches, as next generation drug testing tools, rely on spatially resolved compartments joined together via microgrooves, allowing cell-to-cell interactions, precise control of the 3D cellular and extracellular matrix, and the flow of small amounts of fluids [67]. The integration of functional organ elements onto these structures enables the study of multi-organ interactions and dynamics of drug activities [90]. The rationale is that these emerging in vitro model platforms reconstitute the choroid plexus-mimetic microenvironment more accurately, which enables effective modeling of this tissue for (neuro)therapeutics development and research. Furthermore, the advancement in the 3D printing technology, nanofabrication, integrated sensors, and the versatility of human-derived stem cells have contributed largely to boost the field [91,92]. However, the system setup is sophisticated, time-consuming, and requires specialized equipment, significant resources, and technical skills compared to conventional static models, leading to low-throughput screening capabilities and hindering their broad application. Despite the widespread use of dynamic models, such as cone–plate apparatuses, microporous hollow fibers, and microfluidic-based devices in the generation of in vitro BBB models [93,94], their application and usefulness remain to be adapted for their BCSFB counterparts.

In principle, the high-fidelity microfluidic designs used for the development of BBB-on-chip models, have the potential to be tailored as BCSFB models. They are suitable for co-culturing various cell types, are compatible with high-resolution imaging modalities, allow the monitoring of intracellular and extracellular responses, and have the potential to incorporate patient-specific stem cells towards personalized human brain barrier chips. Accordingly, complex microfluidic BBB devices are described, which can be used as preclinical models to screen brain-targeting drugs, evaluate neurotoxicity, and recapitulate transport processes in the brain under recirculating perfusion [90,95,96]. These BBB-on-a-chip microfluidic models combine the benefits of both in vitro and in vivo models, show significant barrier integrity, and demonstrate a high capacity as drug permeability models using representative drugs and compounds, such as caffeine, cimetidine, doxorubicin, propranolol, antipyrine, carbamazepine, nitrofurantoin, and fluorescent-tagged dextrans [86,90,97,98,99,100].

## 4. Survey of Available Cells

Concerning cell type (origin and species), cell culture-based models of the BCSFB can be based on two different cell varieties, namely cerebral-derived or noncerebral-derived epithelial cells obtained from mammalian and non-mammalian species. Each category can be further divided into primary or continuous cell lines/immortalized cells in terms of proliferative potential. The time-consuming and laborious preparation process of primary and low passage cells has paved the way for employing either cell lines established without genetic manipulation (e.g., from tumors), or laboratory-generated/manipulated immortalized and transfected cell line models. Besides immortalization, these kinds of genetic manipulations can reintroduce the vanished characteristics of the models compared to their respective in vivo tissue. Here, various main cell source details are discussed and summarized in Table 3; however, it is essential to be aware that the selection of cell origin and cell type may have a decisive impact on the experimental costs and outcomes, as well as the reproducibility of obtained results.

### 4.1. Cerebral Originating Cells

The application of human-origin primary cells as in vitro models is restricted due to ethical and technical issues. Therefore, small and large animals have been sources of primary choroid plexus epithelium for various studies. Primary cultures of choroid plexus epithelial cells have been established from various species, including mouse [9,101,102,103,104], rat [16,105,106,107,108], pig [33,58,109,110,111,112,113,114,115,116,117], cow [118,119], sheep [120,121], rabbit [122,123,124], and non-human primates such as rhesus macaque [125]. Canine choroid plexus cells have been isolated as well, though being more challenging compared to other species [16].

Human primary choroid plexus epithelial cells can be obtained from aborted embryos, directly after surgical removal, or postmortem [3,126,127]. However, postmortem-derived human samples may have disturbed viability and functionality depending on the time elapsed after death and could be impacted by the health status of the subject in terms of infections, disorders, injuries, and medication history [3,128,129], and, due to limited applicability for functional assays and usual vectorial transport studies, are gradually abandoned by the researchers in the course of time. HCPEpiC from ScienCell Research Laboratories (also obtainable from other companies) are another human source for primary epithelial cells of the choroid plexus [130,131]. According to the manufacturer, further expansion of these cell populations for 15 doublings is guaranteed. However, multiple concerns are still inherited by this commercially available culture in terms of its origin, nature, and morphology [3].

The experimental procedure to isolate primary choroid plexus epithelial cells from different species and establish a pure cell culture devoid of contaminating cell types has been described comprehensively elsewhere and is not brought up here for the sake of brevity [9,132,133]. Principally, the freshly isolated primary cells retain the differentiated properties of the choroidal epithelium and exhibit a myriad of morphological- and biochemical-desired properties, yet the disadvantages are a small culture size, a limited proliferative potential, and a diminished viability over time after dissection. Therefore, prompt transfer to a suitable in vitro setting is crucial for establishing viable cultures [9]. Likewise, their purity, functionality, recovery, and survival rate explicitly rely on the animals’ health status, isolation approaches/techniques, and applied cultivation conditions [128].

Despite the outstanding contribution of primary cells in comprehensive studies of transport mechanisms and the molecular pharmacology of therapeutics, their potential to be exploited in high-throughput screenings of CNS-acting drugs is rather limited. Accordingly, spontaneous continuous cell lines, immortal/tumor cell lines, and immortalized cells overcome this issue inherited by primary cells and can be propagated easier and with minor limitations.

Z310 and TR-CSFB cells, as rat immortalized cell lines carrying the simian virus 40 large T-antigen gene, are well-characterized choroid plexus epithelial cells that have found their way as suitable cells [25,134,135,136,137,138,139,140]. TR-CSFB cells (five cell lines, TR-CSFB 1–5) exhibit a polygonal-shaped morphology comparable to primary cultured rat choroid plexus epithelial cells. Immunohistochemically, the TR-CSFB cell lines express TTR, apically located Na^+^, K^+^-ATPase, and the efflux transporters ABCB1/MDR1a, ABCC1/MRP1, and ABCG2/BCRP [141]. However, it has been reported that TR-CSFB cells seeded on culture inserts develop a net TEER of only approximately 50 Ω⸱cm^2^. The in vitro transepithelial electrical resistance of the Z310 cells monolayer is declared to reach 150–200 Ω⸱cm^2^ [142]. According to Strazielle and Ghersi-Egea [59], Z310 cells do not affirm a high degree of structural diffusion characteristics indispensable for an eligible BSCFB model and to investigate transepithelial transport processes. Upon culturing on permeable filters, the paracellular diffusion barrier exhibited by Z310 cells is unsatisfactory compared to that of pig counterparts [135,143].

Murine choroid plexus carcinoma cell lines ECPC3 and ECPC4 are isolated from transgenic mice harboring the viral simian virus 40 large T oncogene under the transcriptional control of an intronic enhancer region from the human immunoglobulin heavy chain gene. These cells have shown acceptable stability in culture in a time span of a year [144,145]. The SV11 is another mouse choroid cell line obtained from transgenic mice expressing the SV40 antigen in the choroid plexus [146,147]. The latter has not been characterized in terms of choroidal differentiation status.

The PCP-R cell line has been established based on primary porcine choroid plexus epithelial cells (PCPEC). The cell line exhibits a regular polygonal pattern, expresses junctional proteins, and develops morphologically dense TJs. When cultured on cell culture inserts, this cell line exhibits characteristic barrier properties of TEER above 600 Ω × cm^2^ and a restricted permeability for macromolecular paracellular markers [143,148].

SCP, a sheep choroid plexus epithelial cell line, is another alternative option on hand [13,149,150,151,152,153,154]. A variant of this ovine finite cell line (prepared from brain choroid plexus of *Ovis aries*) is listed in the American Type Culture Collection (ATCC) as cell line SCP No. CRL-1700^®^, or as catalog number 89101302 in the European Collection of Authenticated Cell Cultures (ECACC). However, its applicability as a BSCFB model remains to be validated, since the establishment of junctional complexes and diffusion barrier characteristics essential for a BCSFB model is still an undetermined field for this cell line [59].

Human choroid plexus epithelial papilloma (HIBCPP) cells have been isolated by Ishwata et al. from a 29-year-old Japanese woman, being spindle, oval, and polygonal in shape with neoplastic and pleomorphic features [122]. The cell line prevails over the ethical and technical challenges with primary human cells, and thanks to its human origin, negates any species differences in studies. The advantage of this cell line is that it provides an inexhaustible source of proliferating cells, while preserving the differentiation properties after consecutive passages. On the other hand, the contact inhibition has possibly vanished in these tumor cells leading to disturbed basolateral/apical orientation due to potential overgrowing in multiple layers; nevertheless, there have been reports of establishing optimized protocols endeavored to address this drawback [54].

Other cells, derived from a fragment of a fourth-ventricle choroid plexus papilloma (originated from a 28-year-old male patient) [155], and the CPC-2 cell line, resected from a choroid plexus carcinoma in the left cerebral hemisphere of a 2-month-old boy [156,157,158], have also been reported. The usefulness of these two latter cell lines as a tight and impermeable model of the BCSFB is improbable and not validated.

An immortalized human choroid plexus endothelial cell line (iHCPEnC), expressing pan-endothelial markers and presenting characteristic plasmalemma vesicle-associated protein-containing structures has been generated by Muranyi et al. using transduction of primary human choroid plexus endothelial cells with the human telomerase reverse transcriptase. The resulting cell line grows as a monolayer with contact inhibition and is regarded as invaluable for the generation of in vitro BCSFB co-culture models, as well as contribution to the clarification of the choroid plexus endothelial–epithelial interplay [65].

### 4.2. Noncerebral-Based Cells (Surrogate Models)

Noncerebral-originating cells are considered surrogates in blood–CSF barrier modeling. Madin-Darby canine kidney (MDCK and MDCKII) cells, human colonic epithelial cell line Caco-2 cells, Ralph Russ canine kidney cells (RRCK), Lilly Laboratories Culture-Porcine Kidney 1 epithelial cells (LLC-PK1), and cells transfected with specific efflux transporters or pharmacologic targets can be employed as a surrogate cell-based model to assess the permeability of selected compounds in the presence and absence of overexpressed efflux transporters or to evaluate their mechanism of action and effectiveness [159,160,161,162,163,164,165,166]. The spontaneously immortalized MDCK-MDR1 cell line expressing P-gp/ABCB1, MDCK2-ABCB1 cells expressing ABCB1, and LLC-PK1/BCRP cells expressing the efflux transporter ABCG2/BCRP are examples of surrogate cells, which can be used [167].

## 5. Models Validation Criteria

An effective in vitro model should imitate essential characteristics of an in vivo setting, namely the reproducibility of solute permeability, a restrictive paracellular route, a realistic physiological architecture, the functional expression of transporters/enzymes/receptors, and the ease of culture [55]. This section delineates several standard methods used to qualify in vitro BCSFB models and to evaluate the extent to which the models retain their phenotype and differentiated functions. Thus far, no single model can attain all the strict requirements, but rather the most relevant model configurations for the particular experimental goal should be approached. Certainly, the morphology and quality of in vitro cell-based models can be assured by ultrastructural evaluations, including transmission electron microscopy (TEM) imaging [168]. The determination of the integrity and characteristics of in vitro paracellular barriers can be achieved by evaluating three distinct readily accessible parameters, which are TEER, the permeability for tracer hydrophilic molecules of various but known (predetermined) concentrations and molecular weights, and the qualitative/quantitative expression of TJ proteins and customary markers as well.

### 5.1. Barrier Morphology

Once confluent, an in vitro choroid plexus epithelial cell barrier represents a differentiated polarized morphology, whose ultrastructure can be assessed using standard imaging modalities, including immunofluorescence microscopy and TEM. The latter allows direct visualization of the cells, their organizational structure, and any possible defect or imperfection in the cellular monolayer. Nonetheless, only few studies have used morphology imaging of in vitro cellular barriers as a routine quality validation to assure a closeness to in vivo conditions [168].

### 5.2. Barrier Properties

The reproducible tightness of models can be ascertained by convenient measuring of the TEER. The TEER value is a well-acknowledged measure to appraise the ion permeability of cell layers, reflecting the passive conductance of the TJs to small inorganic electrolytes, and impedance analysis manifests the electrical capacitance of the barrier likewise. Since the TEER can be correlated to the amount and degree of complexity of functional TJs and the expression of microvilli and other membrane invaginations, a high TEER might reflect the resemblance to in vivo situations. As a non-invasive quantitative method, the TEER measurement provides the most selective approach towards evaluating barrier integrity [115,169,170]. Currently, two strategies are employed to measure the TEER, either by using tissue Volt–Ohm resistance meters (voltage-measuring chopstick electrodes, such as the commercially available instruments Millicel-ERS^™^ or EVOM^™^) to measure ohmic resistance or impedance spectroscopy systems (such as the CellZscope chamber-type electrodes from nanoAnalytics) to measure impedance across a broad spectrum of frequencies. There are technical variations between the two methods; while CellZscope yields continuous real-time monitoring of electrical resistance, chopstick electrodes of Volt–Ohm meters can record measurements solely at defined time points. The resulting TEER measures are expressed in an ohm centimeter square (Ω⸱cm^2^) unit. Considering the effect of Ca^2+^ ion concentrations, electrode level of submersion, and electrode position relative to the filter, TEER values can differ across distinct systems.

The analysis of cell monolayers using impedance-based biosensor technologies commercially available (such as xCELLigence technology, which uses proprietary microplates E-Plates) can be regarded as a label-free and real-time measure. The electric impedance-based workflow for monitoring of barrier function shows the advantages of being non-invasive, fast, easy, and real-time compared to end-point assays.

### 5.3. Exogenous Tracer Permeability

Exogenous tracers or inert paracellular flux markers compatible with analytical conditions can provide beneficial information on the permeability status of model barriers towards lipid insoluble organic compounds. However, one should be aware of potential side effects and avoid unnecessary interactions of the tracers with experimental elements. Exogenous tracers come in various physicochemical properties, ranging from proteins and polysaccharides to small polar compounds (such as, but not limited to, mannitol, sucrose, inulin, and dextran) [171]. Most routinely used protein markers are purified plasma proteins such as bovine albumin (~66 kDa), human albumin (~66 kDa), bovine fetuin (~49 kDa), and horseradish peroxidase (40 kDa). It should always be noted that the choice of the protein marker relies upon the biological process being studied and the experimental design. Still, it should be antigenically distinguished from the cells under study. A wide selection of dextran conjugates in a diverse molecular weight range (MW 3, 10, 40, 70, 150, 500, and 2000 kDa) is also available. Owing to satisfactory water solubility, low toxicity, limited immunogenicity, and biologically inertness, dextrans are among the most popular exogenous tracers used to determine barrier tightness.

The permeation of such aptly labeled probes through the cell monolayer as a function of time can be quantitively measured and be relied on as a model validation factor. Using an optimized quantification method (can range from colorimetry, fluorescence spectroscopy, and radioactive scintillation) to measure the concentration of the tracer, the permeability coefficient (P) for each probe can be calculated based on the data obtained from a standard curve and the following equation (based on the assumption that the concentration of the tracer at the donor side remains relatively constant due to the restricted flux and short time frame of the assays).

(1) $$\frac{dQ}{dT}=\left(P\right)\left(A\right)\left(C_{d}\right)$$

where dQ/dT is the slope of the linear segments of the (standard) curve, A is the surface area of the filter, and $C_{d}$ is the tracer concentration in the donor reservoir. The permeability of the cell monolayer ($P_{e}$), generally expressed in cm s^−1^ (or cm min^−1^), can be calculated using the following equation:

(2) $$\frac{1}{P_{e}}=\frac{1}{P_{t}}-\frac{1}{P_{f}}$$

where $P_{f}$ is the permeability of the filters without seeded cells (empty filters) and $P_{t}$ is the total or collective permeability of cells cultured on filters.

Besides providing a BCSFB model quality index and an evaluation of the time-dependent establishment of the barrier, the obtained data from exogenous tracers’ permeability can shed light on the relative contribution of the paracellular compared to transcellular pathways to the overall permeability of an investigational compound. However, attention must be paid that the paracellular marker is chosen appropriately to match the size of the compound [59]. In the following, available dyes and tracers are discussed, and a summarized list of various tracers with their molecular weights and hydrodynamic radii is provided in Table 4.

Dyes: The movement of certain dyes, including trypan blue (an azo dye of 0.96 kDa) and Evans blue (a synthetic dye and a non-specific albumin binder, with a molecular weight of 67 kDa, which produces the Evans blue dye–albumin complex), and phenol red (phenol sulfonphtalein, active transport against concentration gradient) across choroid plexus epithelial cells represents the barrier capacity status of the models [109]. However, since such dyes bind to albumin, their application is more restricted to in vivo models, and since superior and more sensitive tracers are at hand, their use is limited [172,173].

Fluorescent tracers: FITC (fluorescein isothiocyanate), TAMARA (Tetramethylrhodamine), Texas Red^®^, and Alexa Fluor^®^-conjugated dextrans and inulin with various molecular weights are amongst the most popular fluorescent tracers. Conventionally, fluorescently conjugated dextrans, depending on their molecular size, can be used to assess the permeability of models to an ion (low molecular weights variants) and proteins/macromolecules (high molecular weight variants) [174]. Sodium fluorescein (376 Da) is a small molecular-sized marker hydrophilic in nature; with the excitation and emission wavelengths being 494 nm and 525 nm, respectively. Lucifer yellow (absorption 428 nm, emission 533–535 nm), as a small (potassium salt 522 Da, lithium salt 457 Da, and ammonium salt 479 Da) hydrophilic molecule can be used as a tracer for fluid-phase pinocytosis. The assessment of fluorescence intensity can be performed by fluorescence microscopy and/or measurement in a fluorescence plate reader. Working concentrations exhibiting adequate fluorescent signals must be empirically determined for each set of experiments.

Radiolabeled tracers: Small polar compounds radiolabeled with radioactive isotopes can be used, such as [^3^H]-mannitol (182 Da), [^14^C]-sucrose (342 Da), [^3^H]-sucrose, [^3^H]-inulin (~ 5 kDa), [^11^C]-inulin, [^125^I]-albumin (67 kDa), or polyethylene glycol (~4 kDa) [175]. The quantification of radiolabeled tracers is attained by measuring radiation counts by a liquid scintillation system or the quantitative autoradiographic (QAR) method. Despite providing superior sensitivity, radiolabeled markers necessitate special safety precautions and equipment.

Biotin tracers: Besides fluorophores and radioactive isotopes tags, biotin is another compound that can be used in conjugation with tracers to monitor the permeability of in vitro BCSFB models. Biotin-ethylenediamine (287 Da), biotin-dextran, or biotinylated-dextran amine (BDA) with various molecular sizes (3–70 kDa) are exemplary choices. Biotin-labeled exogenous tracers can be visualized both by light- and electron microscopy imaging techniques [172,176,177].

### 5.4. Functional Junctional Proteins and Transporters

Analysis of the expression and localization of junctional proteins and relevant carriers/transporters and their accordance with the choroidal epithelium in vivo can reveal information regarding the quality index of the model. Depending on the individual experimental goal, the characterization of specific markers, enzymes, receptors, adhesion molecules, and specific proteins/polypeptides, can be performed. Immunostaining of cell-type-specific markers and junctional molecules leads to a qualitative confirmation of barrier integrity of an epithelial monolayer.

To assess the efflux transporters’ activity, substrate accumulation assays are exploited. Various efflux inhibitors (such as cyclosporin A and MK-571) are used to perform such substrate accumulation assays, assuming that substrate-inhibitor pairs are appropriately matched for each efflux transporter. In this assay configuration, model cells are incubated with the desired substrate either with or without their respective inhibitors, and at the culmination of the experiment, normalized fluorescence of the cells is calculated. In the case of the functional expression of transporters, the uptake of substrate should be increased in the presence of a corresponding transporter inhibitor [178].

Directional transport: in another experiment variation, transporter activities can be evaluated using directional transport assays, in which inhibitors are only added on the side of the cells grown on cell culture inserts where directional transport is being assessed. Here, again, model cells are incubated with the desired substrate either with or without their respective inhibitors, and the fluorescence signal on the opposite filter side is measured. If efflux transporters are expressed functionally at apical or basolateral surfaces, then the directional transport of substrate in the presence of the corresponding inhibitor is enhanced [178].

### 5.5. Factors Critical to Cell Selection and Culture Conditions

The barrier-forming characteristics and functions of choroid plexus epithelial cells in vitro and their degree of differentiation are closely related to the culture conditions applied. There have been lines of evidence supporting that certain agents and conditions may induce tightening and strengthening of the barrier. The factors critical to cell type selection and culture conditions are discussed in this section.

Serum withdrawal also seems to impact choroid plexus cells to reach the full barrier function. Serum deprivation can tighten the epithelial monolayer and improve the cellular polarity [132,179]. However, the barrier-dismantling effect of serum is believed to be compartment-specific, meaning that it depends on the apical or basolateral exposure of the cell layer to serum. In the case of serum exposure to the apical (comparable to CSF side in vivo) surface of the choroid plexus epithelial cell monolayer, the barrier function is diminished vastly. On the other hand, when serum is applied to the basolateral (corresponding to the stroma/blood side in vivo) surface, the epithelial barrier function is scarcely affected [33,117].

Due to the fact that barrier properties of epithelia are modulated by cAMP-dependent pathways, the presence of membrane-permeable cAMP analogs such as 8-(4-chlorophenylthio)-cAMP (CPT-cAMP) or the adenylate cyclase activator forskolin can have an augmenting effect on TEER values of choroid plexus epithelial cell layers [33,180,181].

Corticosteroids (hydrocortisone, dexamethasone) application to in vitro models may increase the barrier tightness of the epithelial and endothelial cells monolayer. This phenomenon is proposed to be a consequence of the regulation of the expression and distribution of tight junction proteins upon corticosteroids treatment [182]. Dexamethasone, as a synthetic glucocorticoid, has been shown to improve barrier strength and can be exploited as a positive control to investigate the effect of various conditions on the barrier integrity [183,184,185].

## 6. Applications in (Neuro)Therapeutics Development and Research

Several lines of evidence propose that unique transport and barrier attributes of the choroid plexus, in conjunction with the blood–brain barrier, are among the decisive factors establishing cerebral bioavailability of therapeutics and diagnostics. A large number of innovative and highly specific active compounds and investigational pharmaceutical preparations are continuously invented/developed/manufactured during the research and development cycles by the pharmaceutical industry, yet the necessity to discover a practical strategy for the effective delivery of them to the CNS target site remains to be addressed properly. Leveraging a robust and authentic in vitro model of the BCSFB can diminish the time and effort spent on unproductive or redundant development activities by a preliminary assessment of desired physiochemical behavior of an agent toward this barrier. In addition, these model systems can prove helpful in elucidating the nature of permeability and transport mechanisms, with a resolution at the cellular and molecular level, as a focal step during the long journey of neurotherapeutics research and development.

### 6.1. Permeability Screenings and Studies

Any rational drug discovery project dealing with candidates targeted to the brain or requiring exclusion from the CNS to prevent possible side effects should contemplate permeability measurements and prediction studies. Hence, profiling brain/CSF permeability of investigational novel molecular species at preliminary stages of the drug development track is game-changing. To this end, models can predict the CNS permeability of novel or known compounds in relation to both the route and rate.

Most in vitro BCSFB models used for permeability studies/screenings and understanding the pharmacokinetics of CNS active compounds are variants of 2D static bicameral inserts, employed to measure compound movement across the cell monolayer cultured on the porous membrane support. When using these models, a possible disturbance of the mass balance of the initial amount of drug added to the donor chamber with the amount recovered on the opposite reservoir would provide rough evidence of either cellular accumulation or intracellular metabolism of the compound (unspecific adsorption to filter membrane or well plastic should be ruled out). Measuring the concentration of the test compound, using an appropriate method (ranging from chromatography techniques, mass spectrometry, and biological detections methods), as a function of time in one of the compartments and knowing the initial concentration on the opposing compartment yields to the clarification of active transport rate and direction. Subsequently, the permeability coefficient ($P_{e}$) or apparent permeability coefficient ($P_{app}$) (the latter not corrected for permeability of the filter) can be calculated. $P_{app}$ values, expressed in cm s^−1^ or cm min^−1^ unit, can be calculated according to the following equation:

(3) $$P_{app}\;\left[\mathrm{cm}/s\;or\;\mathrm{cm}/\min\right]=\left(\frac{dQ}{dt}\right)\times \frac{1}{C_{0}}\times \frac{1}{A}$$

where dQ/dt is the permeability rate or rate of transfer to the receiver compartment, t is the incubation time (seconds or minutes), $C_{0}$ is the initial concentration in the donor compartment, and A is the filter surface area in ${\mathrm{cm}}^{2}$.

The above equation can be rearranged in terms of the concentration and expressed as follows:

(4) $$P_{app}\;\left[\mathrm{cm}/s\;or\;\mathrm{cm}/\min\right]=\frac{V_{d}\times \Delta M_{r}}{A\times M_{d}\times \Delta t}$$

where $V_{d}$ denotes the volume in the donor compartment in cm^3^; $\Delta M_{r}$ is the total amount of compound in the receiver compartment after time t (seconds or minutes); $M_{d}$ is the donor amount (added at time 0); Δt indicates the incubation time measured in seconds or minutes; and A is the filter surface area in cm^2^.

Eventually, the contribution of both filter and any coating/substrate can be subtracted from the total permeability $P_{app}$ or $P_{t}$ using the following equation leading to $P_{e}$ estimation:

(5) $$\frac{1}{P_{e}}=\frac{1}{P_{t}}-\frac{1}{P_{f}}$$

where $P_{f}$ is the permeability of the filters without seeded cells (empty filters) and $P_{t}$ or $P_{app}$ is the total or collective permeability of cells cultured on the filters.

When applying dynamic microfluidic platforms to measure the permeability of a test (drug)substance added to the basal (mimicking blood side) channel toward the luminal (presenting CSF side) channel, the permeability coefficient $P_{app}$ can be determined using the following equation:

(6) $$P_{app}\left[\mathrm{cm}/s\right]=\frac{C_{l}\times Q}{A\times \left(C_{b}-C_{l}\right)}$$

where $P_{app}$ is the measured or apparent permeability coefficient in the cm/s unit, $C_{l}$ is the luminal concentration, $C_{b}$ is the basolateral concentration, Q is the applied flow rate in the channel (mL/s), the term $C_{l}\times Q\;$(${\dot{m}}_{a}$) represents the mass transport rate (mol/s) across the membrane, and $A\;\left({\mathrm{cm}}^{2}\right)$ is the membrane surface area through which the transport occurs.

The permeability coefficient of a cell layer-free device (blank, or $P_{0}$) can be subtracted from the measured $P_{app}$ to calculate the $P_{e}$ of the model toward the compound, according to the following equation:

(7) $$\frac{1}{P_{e}}=\frac{1}{P_{app}}-\frac{1}{P_{0}}$$

Alternatively, the transport of a given compound can also be calculated from the incremental clearance volume ($\Delta V_{CL}$) for each time point from the following equation [186]:

(8) $$Volume\;Cleared\;\left(\Delta V_{CL}\right)=\frac{C_{a}\times V_{a}}{C_{d}}$$

where $C_{a}$ and $C_{d}$ are concentrations in the acceptor/receiver and donor chambers at sampling time, and $V_{a}$ and $V_{d}$ are acceptor/receiver and donor solutions volumes at sampling time, respectively. Accordingly, the slope of the linear segment of the $\Delta V_{CL}$ versus the time curve leads to the total PS product ($PS_{t}$) in unit of volume/time, assuming that the (drug)substance concentrations in the acceptor/receiver chamber remain small. PS product $PS_{e}$ and finally $P_{e}$ can be obtained from the following equations, leading to the same results as previous equations.

(9) $$\frac{1}{PS_{e}}=\frac{1}{PS_{t}}-\frac{1}{PS_{f}}$$

(10) $$P_{e}=\frac{PS_{e}}{S}$$

where $PS_{t}$ and $PS_{f}$ are the PS products for total model system and cell-free blank filter insert, respectively, and S is the membrane surface area in ${\mathrm{cm}}^{2}$.

An advantage, in the case of measuring the permeability using microfluidic devices compared to static bicameral cell culture filter inserts, is that the drug or substance can be supplied to the basal channel at a constant flow rate and the transported amount can be sampled from the apical/luminal channel with a constant flow rate. Consequently, the assumption that the concentration differences beyond the filter membranes remain constant throughout the experimental read-out is met, albeit in the static bicameral devices this difference decreases over time [187].

The permeation rates of various drugs (ranging from diazepam, propranolol, and morphine, cefadroxil, cyclosporin A, to antiretroviral therapies) [33,188,189], novel central-acting cholinesterase inhibitors (2-phenoxy-indan-1-one derivatives or PIOs) [190], proteins/hormones (thyroxine, leptin, β-amyloids, and TTR) [191], as well as transport kinetics of metals (manganese, iron, and copper) [192] have been assessed effectually with compartmentalized culture insert models.

### 6.2. Transport Mechanisms Studies and (Targeted)Drug Delivery

Thanks to their physicochemical nature, hydrophobic/lipophilic substances of a low molecular weight are capable of free diffusion across cell membranes. In contrast, as mentioned in previous sections, the unrestricted diffusion of hydrophilic chemical species is substantially hindered due to the effective closure and sealing of the paracellular shunt by TJs. Accordingly, access to the CSF is granted exclusively to those compounds that are transported actively by the corresponding transport systems in the plasma membrane of choroid plexus epithelial cells. Theoretically, therapeutic- and diagnostic agents that are effectively and successfully transported by the choroid plexus and remain to some extent unaffected by the metabolizing enzymes and efflux transporters are rapidly distributed throughout the CNS using the bulk flow of CSF. This is due to the fact that at the brain ventricles, the extracellular/interstitial fluid (ISF) and the CSF are separated from each other by the non-barrier/non-restrictive permeable layer of ependymal cells, leading to a direct continuity of these two components and the subsequent free exchange of substances within the extracellular space of CNS [193].

Models expressing the specific transporters found in choroid plexus epithelium could be harnessed to establish whether the permeation of a compound of interest is impacted by a specific carrier system (e.g., P-gp) and to provide comprehensive knowledge on the physiology and modulation of such transporters. From another point of view, several neurological disorders are assumed to be associated with the dysfunction of transporters in the brain, and the detailed region-specific knowledge of transporters and their interaction with investigational therapeutics can prove helpful. In the case that transporters govern the permeability or uptake of a test compound, Michaelis–Menten kinetics can be obtained using nonlinear regression analysis of the concentration dependence influx. In vitro models have proven advantageous and constructive in studies of thyroxine [194], leptin [195], taurine [124], ascorbic acid [112], creatinine [196], and the neuroactive flavonoid resveratrol [197] transport across epithelial cells of choroid plexus. The in vitro BCSFB models have also proven helpful in library screening and identification of cell specific penetrating peptides for the choroid plexus epithelium using phage display techniques [76,198].

Therapeutic and diagnostic agents can pass the BCSFB via the transcellular route by employing one of the possible pathways, which are passive diffusion, facilitated diffusion, or vesicular transfer or transcytosis mechanisms. Generally, cellular uptake mechanisms based on endocytosis/transcytosis are the preferred cell entry route for many compounds. The endocytosis process can be categorized into two broad divisions of phagocytosis and pinocytosis. While the former is restricted to specialized cell types, the latter occurs in all cell types and can be subdivided into macropinocytosis, clathrin-dependent endocytosis (CDE), and clathrin-independent endocytosis (CIE) [199]. Contrary to endocytosis, transcytosis mechanisms are not well understood. Endocytosis and transcytosis across choroid plexus epithelium, studied at the subcellular level, can shed light on mechanisms of targeted delivery of therapeutics across this target site. These studies may rely on endocytosis inhibitors (including, but not limited to, chlorpromazine, genistein, methyl-β-cyclodextrin, and potassium depletion) to provide information concerning the endocytic pathway of compounds under study.

#### Drug Delivery Employing Transport Mechanisms at the BCSFB

Designing novel CNS therapeutics and pharmacologically active candidates that exploit the intrinsic transporting capacity of choroid plexus epithelial cells will prove helpful toward the generation of CNS drug delivery approaches through the BCSFB. Nevertheless, the choroid plexus has received little attention as a potential gateway for drug delivery to the brain, and there remain debates about its eligibility for this aim [200]. An evaluation of the design process effectiveness and rate of successful transport across this barrier can be achieved through preliminary mechanistic and transport studies adopting the available in vitro BCSFB models. Potential transporters that can be targeted to mediate drug delivery to the CNS across the BCSFB belong to the major categories of protein/hormone receptors, solute carriers, and amino acid transporters [201].

Targeting transcytosed receptors (receptor-mediated transcytosis (RMT)): From a pharmacological viewpoint, although most efforts have been concentrated on the BBB, yet the epithelial barrier of the choroid plexus is of considerable importance as a potential target for drug delivery to the CNS, and the subsequent maintenance of effective drug concentrations needed to treat conditions and disorders. Hitherto, transcytosis-mediated transport by interacting with hormone/protein receptors expressed on choroid plexus epithelial cells seems to be the most favorable mechanism exploited for this purpose. The conjugation of BCSFB-penetrating antibodies/peptides with drugs or the engineering of bispecific antibodies, harboring both a therapeutic arm and a BCSFB-transcytosing arm, are among the most promising attempts toward neurotherapeutics-developing pipelines. Other alternatives are vectors based on the native ligand or a fragment of the ligand of the transcytosed receptor.

CNS shuttling of therapeutic and diagnostic macromolecules can be achieved through RMT, an active transport mechanism involving transcytosed receptors, including transferrin receptor (TfR), insulin receptor (IR), insulin-like growth factor receptors (IGF1R and IGF2R), folate receptor FRα, and receptors responsible for lipoprotein transport, namely the low-density lipoprotein receptors (LDLRs) [202,203,204,205,206]. The RMT process is initiated upon ligand binding to the receptors present on the stroma/blood-facing pole of the epithelial cells, consequent ligand/receptor complex internalization and endosomal sorting, followed by ligand release on the apical membrane of the epithelial cells, and receptor recycling back to the basolateral surface. The targeting of transferrin receptors using anti-transferrin receptor antibodies has been the most studied approach so far.

Contrary to solute carrier-mediated transcytosis, targeting an RMT pathway circumvents the size constraints of therapeutic cargos, since it employs a vesicle-based transport rather than a stereoselective carrier. This Trojan horse approach, although being currently studied by many research groups, holds a few technical drawbacks, which need to be addressed. The saturation of transferrin receptors with endogenous transferrin from the circulation requires the transferrin receptor-targeted compound to compete with the natural ligand [207]. As well, the aforementioned RMT targets are also highly and broadly expressed in peripheral tissues and being involved in metabolically critical cellular tasks, arising off-target effects and safety concerns [208,209].

There are reports of enhanced CNS penetration of antineoplastic drugs (methotrexate), peptides (vasointestinal peptide, nerve growth factor), and tracer protein native horseradish peroxidase using anti-transferrin receptor antibody OX26, the mouse monoclonal antibody against the TfR [210,211]. The conjugation of macromolecules to a peptidomimetic monoclonal antibody with an affinity to the human insulin receptor (HIRMAb) has been the topic of multiple experiments using a similar paradigm [212,213]. A HIRMAb-iduronidase fusion protein, valanafusp alpha, is another attempt for efficient brain drug delivery as a therapeutic option for the Mucopolysaccharidosis Type I (MPSI) [214]. The in vitro models of the BCSFB can be helpful to evaluate the transport of anti-TfR antibodies (and obviously other antibodies directed against RMT targets) as vectors for the delivery of a non-lipid-soluble macromolecule into the CNS. The rate of transcytosis can be predicted according to the equations described in the previous sub-sections.

Solute Carrier-Mediated Transcytosis (CMT): Harnessing in vitro BCSFB models, mechanisms pertaining to solute CMT exploited as a drug delivery approach to the CNS, using various solute carrier transporters (SLC) present on apical and/or basolateral membranes of choroid plexus epithelial cells, can be substantiated. In this context, the SLC proteins experience a conformational alteration from an outward to inward-facing orientation and consequently translocate their substrates across the cellular barrier. Generally, small di- and tripeptides can be transferred using this mechanism through the SLC15A family members [202,215]. Although being a promising target, CMT approaches inherit a few disadvantages, such as susceptibility to vector’s stereochemical configuration, rigidity, conjugation regiochemistry, and competition between the drug and original substrate. Furthermore, since SLC-mediated systems are considered portals for the trafficking of small molecule drugs with comparable size and structure to the original ligands, care should be taken upon the cargo’s size to mimic SLC’s original substrates and the vector-drug ligation design to prevent the loss of vector affinity towards the protein carrier [201]. There are few well known examples of drugs that take advantage of this pathway at the BBB, including the anti-parkinsonian drug L-DOPA (through SLC7A5/SLC3A2), antiepileptic agents gabapentin, pregabalin, and valproic acid, choline esterase inhibitor donepezil (through SLC22 subfamily), and N-methyl-D-aspartate receptor antagonist memantine (through SLC22 subfamily). However, the distinct role of active transporters at the BCSFB for this delivery strategy remains to be further clarified [200].

Nano-therapeutics: Nanotherapeutics represent a promising area of CNS drug delivery approaches and have revolutionized the concept of drug delivery across brain barriers with some successful exemplars currently in the clinic [216,217,218]. Nanoparticles (NPs)-based platforms of various size ranges and designs can overcome intact or impaired brain barriers and facilitate efficient CNS drug delivery leveraging transport via multiple mechanisms, including paracellular pathway, cell-mediated transport, adsorptive-mediated transcytosis, RMT, CMT or ligand–receptor interactions [219,220]. Extensive and comprehensive knowledge within the nanomedicine field and available designs of nanocarrier-based drug delivery systems (such as, but not limited to, liposomes, dendrimers, polymeric NPs, polymeric micelles, nanocapsules, lipid-based nanoparticles, inorganic and gold NPs, quantum dots) are summarized elsewhere [219,220,221,222,223,224] and, therefore, are not discussed in detail here.

In principle, all the BCSFB in vitro model systems described so far have the potential to be explored to shed light on the mechanisms of nanotherapeutics interactions with the BCSFB, identify the impact of biological variables on NPs transport across the BCSFB, evaluate the biocompatibility of nanomaterials, help design nanomaterials interacting selectively with the epithelium of the choroid plexus, assess the safety and efficiency of pre-clinical formulations, model the effects of social/stress/immune/environmental factors on NPs transport across BCSFB, and meet the ongoing need of research in neurotherapeutics’ research and development [225,226].

### 6.3. Metabolites/Xenobiotics Transport(er) Regulation

The fate of drugs and xenobiotics metabolites, affiliated to several chemical classes, generated at choroidal epithelial cells and the relative contribution of various efflux pumps present at this barrier can also be investigated using these in vitro models. In addition, the potential effect of multiple metabolites, xenobiotics, and compounds on both influx into and efflux out of the CSF can be studied. Referring to static bicameral device models, efflux transport assays can be performed, and the efflux ratio is estimated by dividing the permeability value in the apical to basolateral (A−B) direction by the permeability value in the basolateral to apical (B−A) direction. With respect to efflux transport assays, the transport of the compounds under study is assessed in the presence of inhibitors of relevant transporter proteins and compared to the non-inhibited conditions. Namely, verapamil, GF120918, PSC833, and N-desmethyl-loperamide as inhibitors of ABCB1/P-gp, Ko143, and fumitremorgin C as an inhibitor of ABCG2/BCRPs, tariquidar and elacridar as inhibitors of both P-gp and BCRP, or MK571 as an inhibitor of various MRPs are examples that can be applied for such experiments [45]. As an alternative strategy, the inhibition of these efflux transporters potentially improves the pharmacokinetics of CNS therapeutic candidates. To this end, the efficacy of a range of compounds (mainly phytoestrogens) as modulators of BCRP/ABCG2 has been evaluated using in vitro BCSFB models [227]. Accordingly, genetic manipulation approaches, such as overexpression, knock-out, or knock-down of specific transporters can be approached.

### 6.4. In Vitro Molecular Verification of Pharmacological Activity

One of the main thrusts behind the development of in vitro BCSFB models has been to shed light on the pharmacological molecular mechanisms of compounds toward this interface. Using cell-based BCSFB models, in vitro activity, potency, and mechanisms of actions of investigational CNS therapeutics and compounds under study can be demonstrated in preclinical drug evaluations. Excerpts of case studies from distinct pharmacological categories describe investigations of the anti-inflammatory activity of synthetic matrix metalloproteinase (MMP) inhibitors [102], and events of receptor activation for drugs of potential abuse and hallucinogens have been studied [228].

Cell cultures of the rat choroid plexus have been used to elucidate the regulation mechanisms and phenomena of various receptors, including 5-hydroxytryptamine_1c_ (5HT_1c_) (using mianserin as an antagonist, or (–)-1-(4-bromo-2,5-dimethoxyphenyl)-2-aminopropane as an agonist) [229].

Investigational anti-viral compounds can be tested in vitro in the BCSFB model platforms. In these experiments, the choroid plexus cells lines could be used as hosts and cellular substrates for viruses to perform a wide range of assays, including the cytopathic effects inhibition assay, the virus titer reduction assay, cytotoxicity assays, for the determination of the effective concentration of compounds under study, and even molecular mechanistic assays. For instance, the activity of nucleoside cytidine analogs as possible anti-HIV compounds was investigated in sheep choroid plexus cells as the cellular substrate for the Maedi-MVV virus [121]. In another study, the potential antiviral activity of teriflunomide against DNA viruses was shown using a choroid plexus epithelial cell model [230].

### 6.5. (Neuro)Toxicological Studies

Harnessing appropriate in vitro BCSFB models, the ever-increasing concern regarding hazardous compounds, such as environmental toxins, metals, pesticides, solvents, as well as the broad field of study of neurotoxicants, can be the target of both exposure (amounts permeated) and impact (effects exerted) investigations. The models have proven valuable in evaluating the influx/efflux of (neuro)toxins, understanding the associated neuroprotective mechanisms, elucidating the alteration of the BCSFB function by toxic compounds, and the involvement of the BCSFB in neuroinflammation [59].

Choroid plexus cells and models have been used to study underlying mechanisms of metal-induced toxicity and the transport of neurotoxicants metals including, but not limited to, mercury (Hg), as presumably the most studied neurotoxic metal, lead (Pb) [231,232,233,234,235], arsenic (As) [236], iron (Fe), manganese (Mn) [237,238,239], copper (Cu) [240], and cadmium (Cd) [241,242].

### 6.6. Pharmacological Interventions at the BCSFB

Transport data acquired through pharmacological interventive measures and modulation by hormones and drugs contribute to understanding the BCSFB homeostatic phenomena and barrier/transporting functions. In addition, establishing approaches for restoring aberrant choroid plexus and CSF dynamics during disease has been of great interest to researchers in pharmacological, neurological, and neurosurgical fields. In this sub-section, major pharmacological mediations used to study this barrier are reviewed.

Carbonic anhydrase inhibitors (acetazolamide, benzolamide): Pharmacological agents of different classes altering CSF dynamics are exploited to study CSF production and secretion. Prototype pharmacological agents to reduce the CSF production rate in the range of 50–100% are inhibitors of carbonic anhydrases, the enzymes responsible for the hydration of CO_2_ to produce carbonic acid (H_2_CO_3_), which spontaneously dissociates into HCO_3_ˉ and H^+^ [23,243,244,245,246]. Acetazolamide is one of the commonly employed drugs in this pharmacological category believed to lessen CSF secretion via its effect on the choroid plexus, and modulation of aquaporin-4 and perhaps aquaporin-1 [247].

Ouabain is a potent inhibitor of the Na^+^-K^+^-ATPase pump and consequently ion and fluid transport. This cardiac glycoside reduces the CSF production rate to a half basal level when placed on the luminal side of the choroid plexus epithelial cells. This aspect along with its neurotoxic side effects hinders its clinical application [11,22,248].

Amiloride, the NHE (basolateral Na^+^-H^+^ exchanger) inhibitor, blocks Na^+^ distribution into the choroid plexus from the blood-facing side [23,249]. This pyrazine diuretic can be exploited to study CSF dynamics.

Loop diuretics (furosemide, ethacrynic acid, and bumetanide) likewise attenuate Na^+^, K^+^, and Cl^−^ transport at choroidal epithelium by inhibiting NKCCs transporters [250,251,252], thereby assisting transport mechanisms studies at this barrier.

Disulfonic stilbene (DIDS) is an anion transporter blocker, inhibiting Cl^−^ transport into the CSF. DIDS is a stilbene-type inhibitor of anion exchangers, which can be used as a pharmacological tool to evaluate the activity of these ion exchangers at the choroid plexus. However, as a disadvantage, its effects are not specific, and a wide range of other transporters can be affected [253,254,255].

Ouabain, acetazolamide, furosemide, bumetanide, and DIDS inhibit CSF secretion from the luminal membrane of choroid plexus. Acetazolamide, DIDS, and amiloride are inhibitors acting on the basolateral/blood-facing membrane [7,256]. In other words, bumetanide and ouabain need to be administered intracerebroventricularly to access their apical target transporters. Such a limitation has suspended their current clinical application; however, effective delivery methods to the CSF would be promising.

The regulation of apical ion channels of choroid plexus by various substances, including serotonin, mianserin, and mesulergine, has been the topic of research [106,257,258]. The integrity of the BCSFB and transepithelial protein transport have been evaluated in the presence of an agonist (GSK1016790A) and an antagonist (HC067047) of the polymodally gated divalent cation channel TRPV4 (transient receptor potential vanilloid 4) [259,260]. Such interventional studies lead to mechanistic clues of the regulation/modulation of receptors and ion channels by investigational compounds.

### 6.7. The BCSFB and Choroid Plexus as a Drug Target in Various Diseases

In this subheading, the role of choroid plexus dysfunction in various diseases and in conditions where itself is a target, for instance, choroid plexus carcinomas, is discussed. Choroid plexus breakdown has been proposed and hypothesized in a wide range of neurological conditions, including aging, Alzheimer’s disease, Parkinson’s disease, epilepsy, stroke, neoplasms, perhaps psychiatric disorders, intracranial hypertension, and certainly varying types of hydrocephalus. Therefore, targeting this tissue may offer opportunities to translate neurotherapeutics from the lab to the clinic, and provide insights toward rational drug therapy to restore barrier function [261,262,263,264].

Strategies to diminish choroidal fluid turnover or to develop efficacious agents, which can manage altered CSF dynamics in disorders, including hydrocephalus, ventriculomegaly, cerebral edema, and intracranial hypertension, can be designed based on the obtained transport data at choroid plexus. In spite of a large body of knowledge of choroid plexus transporters and ion channels that mediate CSF secretion, effective pharmacological agents are sparse, and a gap regarding pharmacological substances capable of interfering still remains.

Supportive evidence proposes the involvement of the choroid plexus in neuroinflammation and neurodegeneration [265,266,267]. Examining the anti-inflammatory potential of a novel or already approved repurposed therapeutics continues to be a subject for major research [268]. Likewise, in vitro cell culture models of the choroid plexus can be approached to answer research questions of the therapeutic efficacy and associated mechanisms of the action/resistance of chemotherapeutic agents to find the right treatment options for the rare brain tumor group of choroid plexus malignancies [147,269].

## 7. Conclusions and Future Outlook

This review set out with the aim of rendering an overview of the up-to-date status of in vitro BCSFB models currently exploited, with a special emphasis on their applicability for research and development of CNS-affecting therapeutics and drug delivery to the brain. Taken together, a suitable model should exhibit the features of the restricted paracellular shunt, the expression of TJ proteins and choroidal markers, and the functional and polarized expression of BCSFB-specific transport mechanisms. In addition, the model should meet the features of availability, convenience, predictability, reproducibility, and extrapolation to human and in vivo settings.

Although a large body of knowledge on transporters, receptors, and drugs pharmaco-kinetics and -dynamics are derived from in vivo animal models and invasive techniques, the fragility of choroid plexus tissue, its small mass, and hardly accessible location along with ethical constraints leads to technical challenges during isolation and further experimental interventions. On the other side, a challenge to reproduce the complexity of the in vivo environment with in vitro models as much as possible still exists. It is generally recognized that the reproducibility of cell-based models can be imperfect, with day-to-day and lab-to-lab inconsistencies being observed. In the case of static monoculture/coculture bicameral devices models, technical challenges may prevail, e.g., the cells can have an incomplete surface coverage or even overgrow and subsequently produce multiple layers instead of monolayers.

Nevertheless, despite all the mentioned shortcomings, in vitro models remain desirable workhorses for routine applications in industrial and academic pharmaceutical studies. Still, it should be emphasized that since no single model can imitate the absolute in vivo physiological environments, for each experimental condition based on the desired outcomes, the question under investigation and the throughput level, a suitable configuration of model system-cell type-validation criteria should be chosen. In other words, by weighing the pros and cons of various platforms, a translationally relevant model of the choroid plexus appropriate for the desired research needs should be selected.

Despite the fact that in vivo, ex vivo, and in situ models (such as models based on isolated choroid plexus in extracorporeal perfusion systems, microdialysis, ventriculocisternal perfusion) may be considered as gold standards and ultimate test systems for exploring drugs transport and pharmaceutically related events, integrated and physiologically related in vitro models are more promising to eliminate the effect of species-specific differences. Furthermore, these in vitro models can be captivating when the aim is to survey transport and other pharmacological mechanisms solely at the choroid plexus in the absence of interfering biochemical effects and traces of the BBB and brain parenchyma. In these model systems, secondary effects originating from drug pharmacokinetics, distribution, and tissue blood flow are eliminated exceedingly.

## Figures and Tables

**Table 1:** Transporters at the BCSFB.

Family	Transporter Function	Members Present on Choroid Plexus Epithelial Cells (Also-Known-as)
SLC1	High-affinity glutamate and neutral amino acids	SLC1A3, SLC1A4, SLC1A5 (ASCT2)
SLC2	Facultative GLUT transporters	SLC2A1 (GLUT1), SLC2A6, SLC2A10, SLC2A12
SLC4	Bicarbonate transporters (anion exchanger)	SLC4A1, SLC4A2 (AE2), SLC4A4, SLC4A5 (NBC4/NBCe2), SLC4A8, SLC4A10, SLC4A11
SLC5	Sodium glucose cotransporters	SLC5A1, SLC5A5, SLC5A6
SLC6	Sodium- and chloride-dependent neurotransmitter transporters	SLC6A4, SLC6A6, SLC6A8 (Crt), SLC6A9, SLC6A11, SLC6A13, SLC6A14, SLC6A15, SLC6A17, SLC6A20A, SLC6A20B
SLC7	Cationic amino acid transporter/glycoprotein- associated	SLC7A1, SLC7A2, SLC7A5 (LAT1), SLC7A6 (LAT2), SLC7A7, SLC7A10
SLC8	Na^+^/Ca^2+^ exchangers	SLC8A1
SLC9	Na^+^/H^+^ exchangers (antiporter)	SLC9A1 (NHE1), SLC9A2, SLC9A6 (NHE6), SLC9A7, SLC9A8, SLC9A9
SLC10	Sodium/bile acid co-transporter family	SLC10A3
SLC11	Proton coupled metal ion transporters	SLC11A2
SLC12	Electroneutral cation-coupled Cl^−^ cotransporters	SLC12A2 (NKCC1), SLC12A4 (KCC1)
SLC13	Human Na^+^-sulfate/carboxylate cotransporters	SLC13A4, SLC13A5
SLC14	Urea transporters	SLC14A2
SLC15	Proton oligopeptide co-transporters	SLC15A2 (PEPT2)
SLC16	Monocarboxylate/monocarboxylic acid transporter family	SLC16A3, SLC16A4, SLC16A6, SLC16A8, SLC16A9, SLC16A10
SLC17	Vesicular glutamate transporters	SLC17A6
SLC20	Type III Na^+^-phosphate cotransporters	SLC20A1, SLC20A2
SLC21/SLCO	Organic anion transporters	SLCO1A5 (OATP1A5), SLCO 1C1, SLCO 2A1 (Pgt), SLCO5A1
SLC22	Organic cation/anion/zwitterion transporters	SLC22A5 (OCTN2), SLC22A6 (OAT1), SLC22A8 (OAT3), SLC22A17, SLC22A18, SLC22A21, SLC22A23
SLC23	Na^+^-dependent ascorbic acid transporters	SLC23A2
SLC24	Na^+^/(Ca^2+^/K^+^) exchangers	SLC24A3, SLC24A4, SLC24A5
SLC25	Mitochondrial carriers	SLC25A1, SLC25A10, SLC25A12, SLC25A14, SLC25A16, SLC25A17, SLC25A18, SLC25A21, SLC25A22, SLC25A26, SLC25A27, SLC25A30, SLC25A32, SLC25A33, SLC25A35, SLC25A37, SLC25A38, SLC25A39, SLC25A45
SLC26	Multifunctional anion exchangers	SLC26A2, SLC26A7
SLC27	Fatty acid transporters	SLC27A1, SLC27A2, SLC27A3
SLC28	Na^+^-coupled nucleoside transporters	SLC28A3
SLC29	Facilitative nucleoside transporters	SLC29A2, SLC29A4 (PMAT)
SLC30	Zn^2+^ efflux transporters	SLC30A3, SLC30A4, SLC30A5, SLC30A6, SLC30A9, SLC30A10
SLC31	Cu^2+^ transporters	SLC31A1, SLC31A2
SLC33	Acetyl-CoA transporters	SLC33A1
SLC35	Nucleoside-sugar transporters	SLC35A1, SLC35A4, SLC35A5, SLC35D2, SLC35E2, SLC35E4, SLC35F1, SLC35F2, SLC35F3, SLC35F5
SLC37	Sugar-phosphate/phosphate exchangers	SLC37A1 (G3PP), SLC37A2
SLC38	Amino acid transporter	SLC38A1, SLC38A3, SLC38A4, SLC38A5, SLC38A11
SLC39	Metal ion transporters	SLC39A4, SLC39A8, SLC39A10, SLC39A11, SLC39A12, SLC39A14
SLC40	Basolateral Fe^2+^ transporters	SLC40A1
SLC41	MgtE-like magnesium transporters	SLC41A1, SLC41A2
SLC43	Na^+^-independent, system-L-like amino acid transporters	SLC43A1, SLC43A2
SLC44	Choline-like transporters	SLC44A3
SLC45	Putative sugar transporters	SLC45A4
SLC46	Folate transporters	SLC46A1, SLC46A3
SLC48	Heme transporters	SLC48A1
SLC50	Sugar efflux transporters	SLC50A1

**Table 2:** Summary of in vitro BCSFB models.

Model and Its Description	Applications	Advantages	Disadvantages	Throughput
2D static bicameral devices (cell culture inserts) or compartmentalized monocultures.	Compartmentalized culturing. Routinely used for permeability studies. Evaluation of (neuro)toxicological profile of investigational compounds. Lead compound identification/optimization phase studies. Structure–activity relationships (SAR) analyses.	Being user-friendly, easy to set up, and low labor intensity. Minimal cost. Used when the isolated study of compounds and epithelial cells interaction is the aim.	2D structure. Generally low TEER values. Too simple to fully replicate key features of the BCSFB. Lack of contact with other cells. Fail to mimic CP microenvironment due to lack of shear stress and blood/CSF flow. Real-time readouts are not easily possible.	Moderate (offers HTS capabilities).
Co-culture models.	Study drug permeability.	Allows co-culture of endothelial cells and other related cells. Takes into account the impact of other elements of the BCSFB. Higher TEER and greater barrier stability.	Lack the fluid flow-induced shear stress. Relatively time-consuming. Higher cost.	Moderate.
3D and organoids.	Search for therapeutic targets. Study interventional strategies to control drugs and substances entry. Evaluation of (neuro)toxicological profile of investigational compounds. Lead compound identification/optimization phase studies. Structure–activity relationships (SAR) analyses.	Human origin cells/tissue can be used. 3D culture model. Reduced re-differentiation. High barrier tightness. Better maintain the tight junction organization compared to bicameral systems. Possibility to be applied for personalized medicine developments by using cells/tissues from a specific donor group. Allow studies on an organ-level in both healthy and diseased conditions.	Not applicable for high-throughput quantitative permeability measurements. Lack the fluid flow-induced shear stress. Complicated procedure with greater skill required. High processing time. Differential process relies upon random and continuous addition of differentiation factors.	Low to medium.
Dynamic models (microfluidic or organ-on-a-chip platforms).	Study drug permeability. Study drug’s Pharmacokinetic elements.	Possibility of integrating imaging systems and sensors with real-time readouts. Human-derived cells or tissues can be used. Contribution of fluid shear stress as an important factor is considered.	Difficult to set up and maintain. High technical prerequisite needed.	Low to medium.

**Table 3:** Summary of available cells lines.

Cell Types	Main Advantages and Disadvantages	Origin	Cells	Species Source
Primary cell cultures	More representative of the in vivo state. But usually expensive and labor-intensive to isolate. Have limited yield. Exhibit difficulty obtaining from human origin.	Cerebral	Pig primary cells, PCPEC	Pig
			Mouse primary cells	Mouse
			Rat primary cells	Rat
			Bovine primary cells	Cow
			Ovine primary cells	Sheep
			Rabbit primary cells	Rabbit
			HCPEpiC	Human
		Non-cerebral	No Reports	
Immortalized and continuous cell lines	Affordable and easily accessible. Commercially available. Easy to culture. But usually do not mimic the native CP. Possibly altered genotype/phenotype after many passages.	Cerebral	Z310	Rat
			TR-CSFB	Rat
			ECPC3	Mouse
			ECPC4	Mouse
			SV11	Mouse
			PCP-R	Pig
			HIBCPP	Human
			CPC-2	Human
			iHCPEnC	Human
		Non-cerebral	MDCK	Dog
			MDCK-MDR1	Dog
			RRCK	*Dog*
			Caco-2	Human
			LLC-PK1	Pig

**Table 4:** Exogenous tracers categorized based on target solute/compound permeability.

Marker		Size	
		Molecular Weight (Da)	Approximate Hydrodynamic Radius (nm)
Markers of Protein/Macromolecules Permeability			
Dyes	Evans blue	960	NR ^1^
	Trypan blue	961	NR
Fluorescent tracers	FITC-dextran 150 kDa	150,000	9.0 ± 0.6
	FITC-dextran 70 kDa	70,000	6
	FITC-dextran 40 kDa	40,000	4.5
	FITC-albumin	67,000	5.4 ± 0.1
Horseradish peroxidase		40,000	5–6
Microperoxidase		1900	3.0
Radiolabeled Compounds	[^125^I]-albumin	~69,000	3.5
	[^14^C]-dextran 70 kDa	~70,000	6
Markers of Solute and Ion Permeability			
Ionic Lanthanum		138.9	0.12
Sodium Fluorescein		376	0.45
Lucifer Yellow		457	0.42
Biotin ethylenediamine		286	NR
FITC-dextran 3kDa		3000	1.4
Radiolabeled Compounds	[^14^C]-α-Aminoisobutyric acid	103	NR
	[^14^C]-Sucrose	342	0.46
	[^3^H]-mannitol	182	0.36
	[^14^C]-Methotrexate	455	NR
	[^14^C]-Inulin	5000	1.3

^1^ Not Reported.

## Abbreviations

2D	two dimensional
3D	three dimensional
5-HT	5-hydroxytryptamine
ABC	ATP-binding cassette
ADME	absorption, distribution, metabolism, excretion
ANP	atrial natriuretic peptide
ATPase	adenosine triphosphatase
AVP	arginine vasopressin
BBB	blood–brain barrier
BCRP	breast cancer resistance protein
BCSFB	blood–cerebrospinal fluid barrier
CDE	clathrin-dependent endocytosis
CIE	clathrin-independent endocytosis
CMT	carrier-mediated transport
CNS	central nervous system
CP	choroid plexus
CSF	cerebrospinal fluid
EHs	epoxide hydrolases
ESCs	embryonic stem cells
FITC	fluorescein isothiocyanate
FMOs	flavin-containing monooxygenases
FPRL1	formylpeptide receptor-like 1
GSTs	glutathione S-transferases
HTS	high throughput screening
IGF	insulin-like growth factor
IGFR	insulin-like growth factor receptor
iPSCs	induced pluripotent stem cells
IR	insulin receptor
ISF	interstitial fluid
JAMs	junctional adhesion molecules
LDLR	low-density lipoprotein receptor
LLC-PK1	Lilly laboratories culture-porcine kidney 1
LRP	LDLR-related protein
MAGUKs	membrane-associated guanylate kinase-like homologues
MDCK	Madin-Darby canine kidney
MDR	multidrug resistance protein
MMP	matrix metalloproteinase
MPS	Mucopolysaccharidosis
MRP	multidrug resistance-associated protein
NPs	Nanoparticles
NHE	Na^+^-H^+^ exchanger
PC	polycarbonate
PCPEC	porcine choroid plexus epithelial cells
PET	polyethylene terephthalate
P-gp	P-glycoprotein
PIOs	2-phenoxy-indan-1-one
PTFE	polytetrafluoroethylene
QAR	quantitative autoradiographic
RMT	receptor-mediated transcytosis
RRCK	Ralph Russ canine kidney
SAR	structure–activity relationships
SLC	solute carrier
TAMARA	tetramethylrhodamine
TEER	transepithelial electrical resistance
TEM	transmission electron microscopy
Tf	transferrin
TfR	transferrin receptor
TJ	tight junction
TTR	transthyretin
UGTs	UDP-glucuronosyltransferases
ZO	Zonula occludens
